# Crickets in the spotlight: exploring the impact of light on circadian behavior

**DOI:** 10.1007/s00359-023-01686-y

**Published:** 2024-01-22

**Authors:** Keren Levy, Anat Barnea, Eran Tauber, Amir Ayali

**Affiliations:** 1 School of Zoology, Tel Aviv University, 6997801 Tel-Aviv, Israel; 2 Department of Natural Sciences, The Open University of Israel, 4353701 Ra’anana, Israel; 3 Department of Evolutionary and Environmental Biology, Institute of Evolution, University of Haifa, 3103301 Haifa, Israel; 4 Sagol School of Neuroscience, Tel Aviv University, 6997801 Tel-Aviv, Israel

**Keywords:** Artificial light at night (ALAN), Light pollution, Chronobiology, Circadian clock, *Gryllus bimaculatus*

## Abstract

Crickets serve as a well-established model organism in biological research spanning various fields, such as behavior, physiology, neurobiology, and ecology. Cricket circadian behavior was first reported over a century ago and prompted a wealth of studies delving into their chronobiology. Circadian rhythms have been described in relation to fundamental cricket behaviors, encompassing stridulation and locomotion, but also in hormonal secretion and gene expression. Here we review how changes in illumination patterns and light intensity differentially impact the different cricket behaviors as well as circadian gene expression. We further describe the cricket’s circadian pacemaker. Ample anatomical manipulations support the location of a major circadian pacemaker in the cricket optic lobes and another in the central brain, possibly interconnected via signaling of the neuropeptide PDF. The cricket circadian machinery comprises a molecular cascade based on two major transcriptional/translational negative feedback loops, deviating somewhat from the canonical model of *Drosophila* and emphasizing the significance of exploring alternative models. Finally, the nocturnal nature of crickets has provided a unique avenue for investigating the repercussions of artificial light at night on cricket behavior and ecology, underscoring the critical role played by natural light cycles in synchronizing cricket behaviors and populations, further supporting the use of the cricket model in the study of the effects of light on insects. Some gaps in our knowledge and challenges for future studies are discussed.

**Supplementary Information:**

The online version contains supplementary material available at 10.1007/s00359-023-01686-y.

## Introduction

In this review we seek to provide a perspective on the contribution of the cricket as a model insect to the study of the effects of light on animal physiology, behavior, and ecology. We first briefly summarize the general importance of natural light cycles and of light as a *zeitgeber* stimulus. We then present crickets as models for studying chronobiology and the effects of light, including some historical perspectives. This is followed by discussing research into the location of the cricket circadian pacemaker, and current updates regarding the circadian clock machinery. Next, we present the topic of artificial light at night (ALAN) and review studies of the impact of ALAN on crickets and on their circadian behavior. We conclude with a short discussion and some future potential research directions.

## Light and the circadian system

In most organisms, the diel cycles of light and darkness constitute crucial cues for the temporal organization of behavior (Pittendrigh 1961; Aschoff 1981). Compared to other environmental variables that show diurnal fluctuations (e.g., temperature, humidity), the light-dark diel cycle is the most reliable cue for entraining an animal’s circadian system, i.e., for synchronizing daily activity patterns, behavior, and physiological processes such as hormonal secretion and gene expression, to external cues and the surrounding environmental conditions (Helfrich-Förster 2020).

Light effects are manifested via the circadian clock—an endogenous cell-autonomous pacemaker that generates rhythms with a periodicity close to 24 h (Pittendrigh 1961). Through the process of entrainment, light cues are used to adjust the circadian period to exactly 24 h. Importantly, upon entrainment, the pacemaker assumes a new fixed phase relative to the light-dark cycle (Mrosovsky 1999; Helm et al. 2017). This phase represents the activity preference of the animal, whether diurnal (day active), nocturnal (night active), or crepuscular (active during twilight) (Aschoff and von Goetz 1988; Helm et al. 2017). The extent to which the circadian clock is entrained depends on the properties of the light stimulus (intensity, spectrum) and on the light sensitivity of the pacemaker, which vary throughout the day (Daan and Aschoff 2001).

**Fig. 1:**
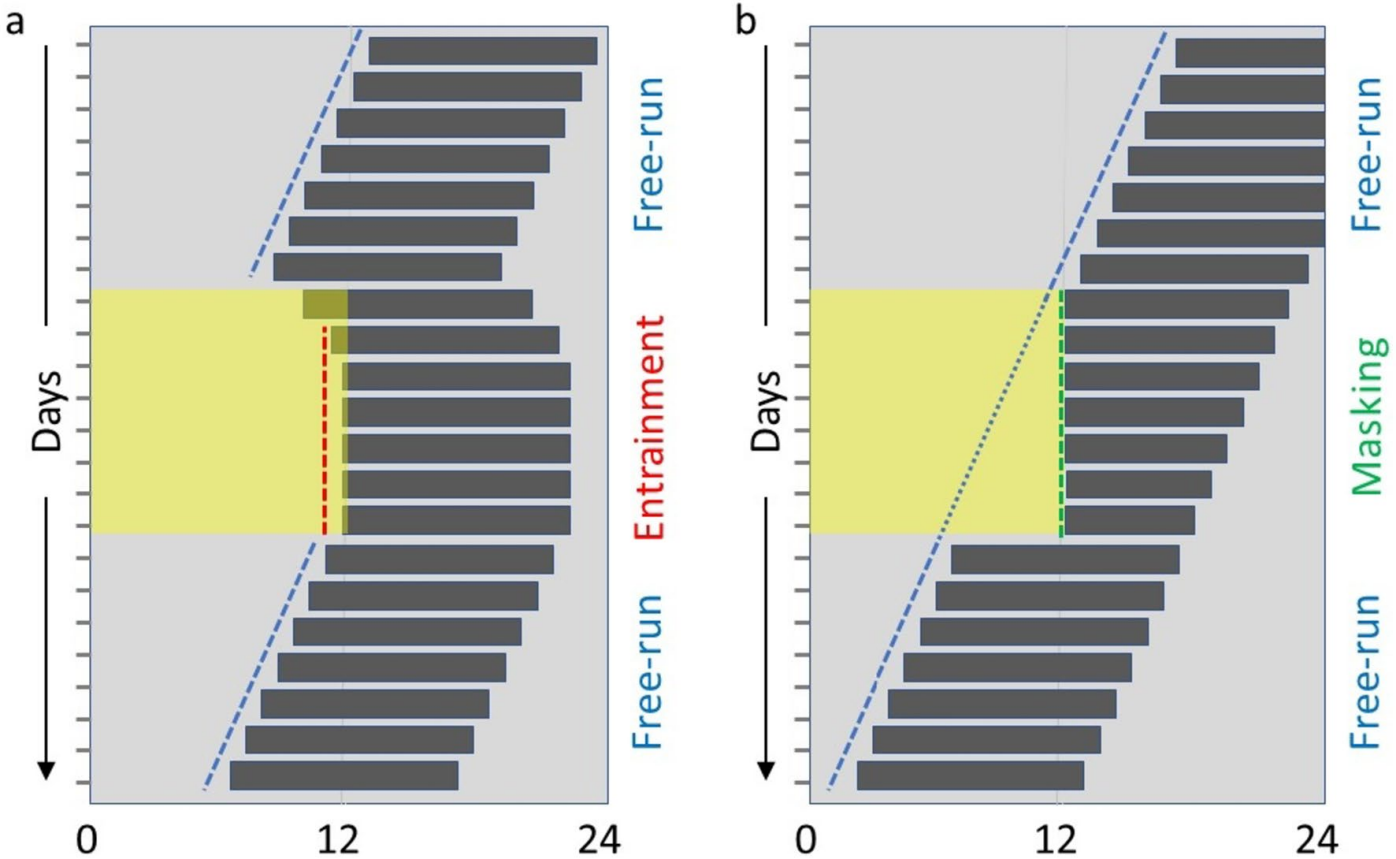
A schematic representation of the possible effects of light stimuli on the behavioral patterns of animals. **a** Entrainment to the light stimulus—the effect persists also after return to constant darkness, **b** masking—instantaneous and transient—not persisting after return to constant darkness. Grey bars represent activity; yellow and light grey indicate light and darkness, respectively

Entrainment is best studied in the laboratory. The rhythmic behavior of the animal can be tested under constant conditions (e.g., continuous darkness) that allow the pacemaker to ‘free-run’ (Fig. 1). Upon switching to light-dark conditions the pacemaker is entrained and runs with a 24 h period (Fig. 1a). Light may have an additional, direct effect, called *masking* (Fig. 1b) on certain behaviors. This does not involve the circadian clock and overrides its rhythm (Mrosovsky 1999). The masking response is instantaneous and transient (Fig. 1b).

Light pulse experiments in the laboratory provide a means by which to characterize the circadian light sensitivity. Natural variation in light sensitivity can take place at the species, population, or individual level. When testing the light effects, the specific properties of an animal’s visual system e.g., its photoreceptors, spectral sensitivity, and visual acuity (Warrant and Nilsson 2006; Land and Nilsson 2012; Van Der Kooi et al. 2021), as well as its visual sensory processing (e.g., Blum and Labhart 2000; Mappes and Homberg 2004; Okamoto et al. 2001) should be taken into consideration, in addition to the nature of the specific stimulus, its duration and intensity.

## The cricket as a model organism in chronobiology studies

For more than a century, crickets (Order: Orthoptera) have served as model organisms for biological research (Regen 1913; Fabre et al. 1921), including behavioral and neurobiology studies. Crickets have been much utilized in studies of song generation (Bentley and Hoy 1974; Huber et al. 1989; Jacob and Hedwig 2019), auditory processing (Zorović and Hedwig 2013; Schöneich 2020), acoustic communication (Libersat et al. 1994; Hall and Robinson 2021), sexual selection (Regen 1913; Simmons 1986; Simmons and Richie 1996; Tyler et al. 2015; Benavides-Lopez et al. 2020), aggression (Stevenson and Rillich 2012; Rillich and Stevenson 2019), and escape behavior (Tauber and Camhi 1995), as well as studies of learning (Matsumoto 2022) and the neuronal and behavioral responses to polarized light (Labhart et al. 1984; Labhart and Keller 1992; Labhart 1999). Their much-studied physiology and well-characterized behavioral repertoire have enabled additional research of crickets as an important model in chronobiological research (Horch et al. 2017; Numata and Tomioka 2023).

**Fig. 2:**
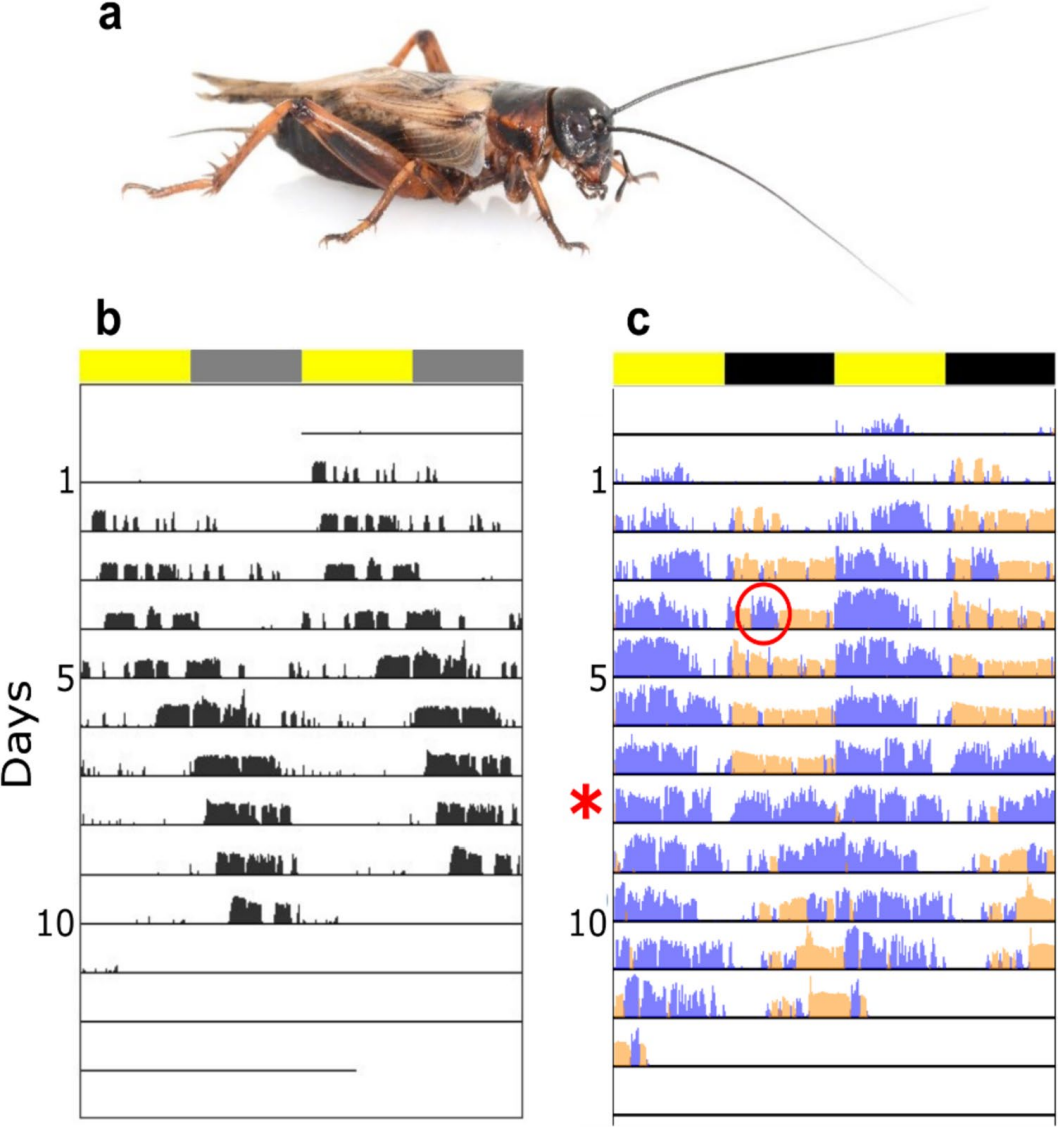
Actograms showing the rhythmic behavioral patterns of *G. bimaculatus* male crickets (**a**). **b** Free-run behavior induced by exposure to artificial light at night (adapted from Levy et al. 2021), and **c** simultaneous record of nocturnal stridulation (orange) and diurnal locomotion (blue) behaviors. A nocturnal light pulse induced negative and positive masking of stridulation and locomotion, respectively (red circle, line 4). Yellow bars indicate day, and black or gray bars indicate night, while rows indicate consecutive days. The red asterisk indicates a switch to a free run pattern in both behaviors (day 8) following constant illumination

Orthopterans encompass both diurnal and nocturnal species and occupy a wide range of habitats. This is reflected in a variety of visual properties and visual sensitivity levels, including color vision, which has only been described to date in a small number of species (Alaasam et al. 2021; Van Der Kooi et al. 2021). The nocturnal, ground-dwelling field cricket, *Gryllus bimaculatus* (Fig. 2a), possesses the following types of visual receptors: UV (peak: 332 nm), blue (peak: 445 nm), and green (peak: 515 nm) (Zufall et al. 1989; Henze et al. 2012), while, like many insects, lacking a red receptor. The cricket thus may be capable of trichromatic color vision. The blue receptor was found to be mostly involved in polarized light vision (Labhart et al. 1984; Herzmann and Labhart 1989). Polarized light vision, thoroughly investigated in several cricket and locust species, is utilized by many insects for orientation and spatial navigation, using the sky’s compass information (Wehner 1984; Brunner and Labhart 1987; Barta and Horváth 2004; Mappes and Homberg 2004; Henze and Labhart 2007).

As further discussed below, the green-sensitive (long wavelength) opsin (*OpLW*) was described in *G. bimaculatus* crickets as the major circadian photoreceptor molecule, which initiates the cascade responsible for their circadian entrainment (Komada et al. 2015). Hence, the crickets’ compound eye is involved in both the visual and the circadian pathways. Overall, the visual system and signal processing of these crickets (*Gryllus* sp.) are well adapted to life under near-dark conditions (Zufall et al. 1989; Sakura et al. 2003; Frolov et al. 2014; Frolov and Ignatova 2020). The cricket’s high sensitivity to very low light intensities has made it a useful model for research into the various effects of light, including in chronobiological research.

Courtship behavior of male crickets consists species-specific calling songs produced by rubbing their front wings together (stridulation; see supplementary video S1). More than a century ago, crickets were already being reported to stridulate just after sunset and during the night in order to attract females for reproduction, thus presenting a clear diurnal behavior (Fabre et al. 1921; see also: Loher et al. 1993; Simmons 1988, 1986). The first researcher to introduce the use of crickets into modern chronobiology experiments was Lutz (1932), who recorded the house cricket’s (*Acheta domesticus*) circadian locomotion activity under daylight conditions and in subsequent constant darkness. The cricket showed clear diurnal rhythms with a nocturnal activity peak in the first half of the night, a pattern that remained consistent even under conditions of constant darkness (Lutz 1932).

Since Lutz’s pioneering study, both stridulation and locomotion activities have been widely examined in order to assess the effects of changes in illumination patterns on cricket behavior. The expression of two or more different circadian behaviors (e.g. stridulation and locomotion), can be monitored simultaneously (Fig. 2c), thus enabling the assessment of differential responses to the same light stimulus (Sokolove 1975; Fergus and Shaw 2013; Levy et al. 2021, 2023a). Simultaneous monitoring of locomotion and stridulation behaviors in male *Teleogryllus commodus* and *G. bimaculatus* crickets in the laboratory revealed different phases of the circadian rhythm for each of these behaviors, with stridulation being nocturnal and locomotion either occurring at night or during the day (depending on the species and the light intensities used; Germ and Tomioka 1998; Levy et al. 2021, 2023a; Okamoto et al. 2001; Sokolove 1975; Tomioka and Chiba 1982a). Moreover, changes in illumination patterns were reported to differentially affect both these circadian behaviors, inhibiting stridulation while increasing locomotion behavior (Levy et al. 2023a). Similarly, light-induced changes in various activity patterns (Sokolove 1975; Abe et al. 1997) were monitored parallel to changes in circadian gene expression (Moriyama et al. 2009; Fergus and Shaw 2013; Tokuoka et al. 2017; Levy et al. 2022), thus connecting multiple behavioral, physiological, and transcriptional responses in order to obtain a better understanding of the multifaceted effects of light on the crickets.

Crickets, consequently, offer several specific advantages as model organisms in research into chronobiology and the effects of light (Horch et al. 2017; Numata and Tomioka 2023), including: (1) a short lifecycle and ease of rearing in the laboratory under various controlled conditions; (2) circadian patterns—expressed in a variety of measurable traits, such as locomotion, stridulation, and hormonal secretion, which can all be monitored either separately or simultaneously; (3) sensitivity to changes in illumination levels and patterns; and (4), a well-described circadian clock machinery, including description of many of the related genes.

## The location of the crickets’ circadian pacemaker

Initial insights into crickets’ circadian mechanisms were provided by experiments in which the compound eyes and/or the ocelli were blacked out. The results of these experiments indicated the involvement of the compound eyes or the optic lobes in generating behavioral rhythms (Nowosielski and Patton 1963). Cyclic oscillations were then described in the brain of *A. domesticus*, including acetylcholinesterase levels and RNA synthesis (Cymborowski and Dutkowski 1969), as well as in the ultrastructure of the medial neurosecretory cells (Dutkowski et al. 1971). An important experiment was conducted by Cymborowski and Brady (1972), repeating and reconfirming previous, yet somewhat disputed, studies, and involving a pair of *A. domesticus* crickets, each running on a different circadian phase. The two crickets were waxed together with interconnected hemocoels, one serving as a “donor” and the other (with its brain removed), serving as a recipient. The experiment resulted in a locomotion activity rhythm in the recipient cricket identical to that of the donor. This study unequivocally demonstrated that the locomotion circadian rhythm is induced and controlled by a humoral factor originating in the cricket’s brain (Cymborowski and Brady 1972; Brady 1974). This was further confirmed by transplanting the brain of a rhythmically light-entrained cricket into the abdomen of an arrhythmic one, which resulted in the production of a circadian rhythm in the recipient cricket, thus again indicating the existence of a circadian pacemaker in the brain (Cymborowski 1981).

The role of the compound eyes was again demonstrated by various anatomical manipulations, such as severing the pathways between the ocelli and the brain, between the compound eyes and the optic lobes, and between the optic lobes and the brain, leading Sokolove and Loher (1975) to the conclusion that the photic circadian signal is delivered through the compound eyes, rather than the ocelli (Sokolove and Loher 1975). Further investigations into the role of the eyes and optic lobes in the cricket’s circadian clock were carried out by Tomioka and colleagues in the cricket *G. bimaculatus.* A circadian electroretinogram (ERG) rhythm recorded from the cricket’s compound eyes was found to persist even after severing the optic tract and isolating it from the central nervous system (Tomioka and Chiba 1982b, 1985). Additionally, circadian neuronal activity and daily oscillations in serotonin levels were observed in the lamina-medulla complex of the cricket’s optic lobe and were found to increase towards and during the nighttime (Tomioka and Chiba 1986; Tomioka et al. 1993), indicating the optic lobes as the location of the main circadian pacemaker (Tomioka and Chiba 1982b, 1985, 1989, 1992). This finding was in agreement with multiple studies in different cricket species that had shown an arrhythmic pattern of stridulation behavior following optic tract severance (Nowosielski and Patton 1963; Loher 1972; Rence and Loher 1975; Sokolove and Loher 1975; Tomioka and Chiba 1986; Okada et al. 1991; Abe et al. 1997; Okamoto et al. 2001; Tomioka and Matsumoto 2015; Kutaragi et al. 2018), thus indicating the essential role of the optic lobe in light entrainment. Several studies, however, reported retained rhythmic activity even following optic tract severance (Rence and Loher 1975; Tomioka 1985; Stengl 1995), supporting the notion of an additional oscillatory center, presumably located in the central brain (responsible for releasing the humoral factors synchronizing the circadian rhythms).

## The crickets’ circadian clock machinery

**Fig. 3:**
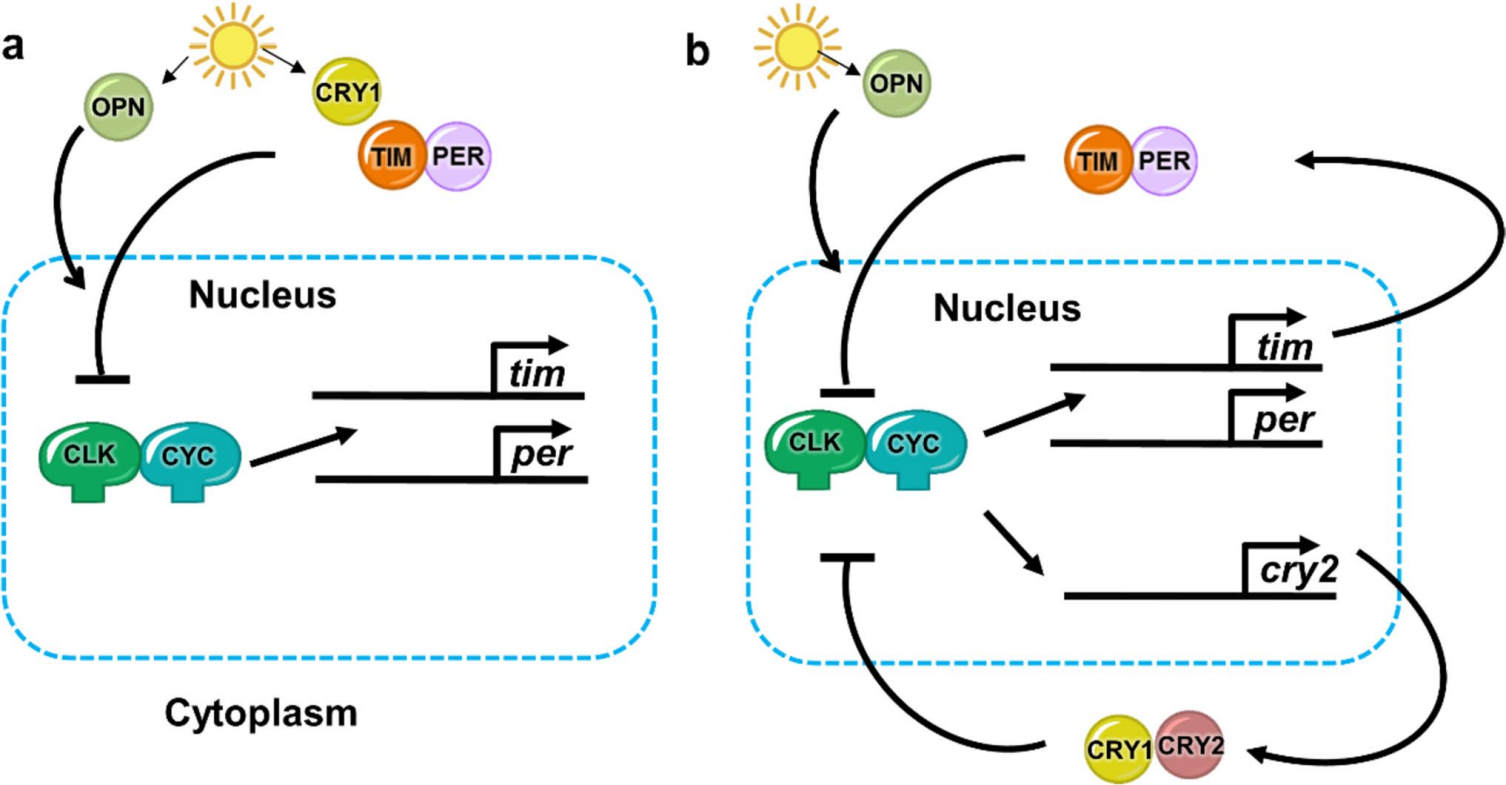
The circadian transcription-translation negative feedback loop in fruit-flies and crickets (simplified schematic). **a** In *Drosophila*, CLK:CYC drive the expression of TIM and PER, which translocate to the nucleus and inhibit their own transcription. CRY1 serves as a blue-light photoreceptor that drives the light-dependent degradation of TIM. Photoreceptor opsins in the retina (OPN) also contribute to the light input. **b** In crickets, CRY1 and CRY2 serve as transcription repressors. Light entrainment is driven by a green sensitive opsin. See text for more details. The cricket schematic is adapted from Tokuoka et al. (2017)

The circadian pacemaker consists in an intricate network of so-called “clock genes” and their corresponding protein products. This network, which was first identified in the fruit fly *Drosophila*, is evolutionarily conserved, although studies in crickets and other insects have revealed different variations (Fig. 3). The fundamental constituents of the core circuit comprise *period* (*per*), *timeless* (*tim*), *Clock* (*Clk*), and *cycle* (*cyc*) genes (see a recent detailed review in Numata and Tomioka 2023). Within this system, *Clk* and *cyc* encode the transcription factors CLOCK (CLK) and CYCLE (CYC), which form a heterodimer. This heterodimer subsequently activates transcription of the *per* and *tim* genes (Allada et al. 1998; Rutila et al. 1998). The transcripts of *per* and *tim* are then translated into the corresponding protein entities (PER, TIM). These proteins engage in heterodimerization, translocate back to the cell nucleus, and effectively inhibit their own transcription by blocking the transcriptional activity of CLK/CYC (Sehgal et al. 1994). With the gradual reduction of PER and TIM, inhibition is removed and a new cycle commences. This intricate interplay establishes a negative feedback loop, giving rise to the characteristic 24-h oscillation (Fig. 3).

In *Drosophila*, *cryptochrome* (*cry*) encodes a blue-light photoreceptor that contributes to light resetting the clock through its interaction with TIM (Ceriani et al. 1999). In contrast, in crickets, two paralogous genes are present: *cry1* (or *Drosophila*-type *cry*) and *cry2* (or mammalian-type *cry*). RNAi-mediated knocking down of *cry1* or *cry2* did not prevent photic entrainment, indicating that neither CRY1 nor CRY2 are circadian photoreceptors (Tokuoka et al. 2017). However, dual RNAi of *cry1* and *cry2* repressed CLK/CYC transcriptional activity.

In crickets, the molecular pathway involves two major transcriptional/translational negative feedback loops: namely, the *per/tim* loop and the *cry1/cry2* loop (Fig. 3b; Tokuoka et al. 2017). The two loops can oscillate independently of one another, and control for circadian rhythm generation and rhythmic expression of the other clock-related genes. The *cry1/cry2* loop plays a role in fine-tuning and even resetting the clock to the day/night cycle. The two negative feedback loops are coupled in that both play a role in suppressing the transcription mediated by the *clk/cyc* complex (Moriyama et al. 2012; Uryu et al. 2013; Tokuoka et al. 2017).

Light-induced resetting of the clock is mediated by the green-sensitive opsin (*OpLW*) (Komada et al. 2015). In addition, the proteins encoded by *par domain protein 1 (pdp1)* and *c-fosB* are upregulated by light-induced neurotransmitter release. These two proteins affect the above feedback loops through modulation of *cry* and degradation of *tim*, followed by resetting the phase of the *per/tim* loop (Fig. 3; Kutaragi et al. 2016, 2018; Tokuoka et al. 2017; Tomioka and Matsumoto 2019; Narasaki-Funo et al. 2020).

In several insect species, including *Drosophila*, *Rhyparobia*, and *Rhodnius*, specific neurons within the accessory medulla have been identified as circadian pacemakers (Helfrich-Förster 2003; Schneider and Stengl 2005; Vafopoulou et al. 2007; Shafer and Yao 2014; Hamanaka et al. 2022). However, the evidence for such pacemaker cells in crickets is less clear. In *Drosophila*, the neuropeptide Pigment Dispersing Factor (PDF) is the main neuromodulator of the circadian clock network (Renn et al. 1999; Helfrich-Förster et al. 2000; Yoshii et al. 2012) and is expressed in the clock neurons in the brain. In crickets, PDF has been found in the central brain, in the optic lobe, and in the cerebral lobe, presenting a daily cycle that peaks nocturnally (Homberg et al. 1991; Abdelsalam et al. 2008). PDF was suggested to be involved in phase regulation of the daily rhythm (Singaravel et al. 2003) and, importantly, to increase the light responsiveness in the neurons that couple the two optic lobes (medulla bilateral neurons, MBNs) (Saifullah and Tomioka 2003). Serotonin was also found to phase-shift the circadian clock in the optic lobe (Tomioka 1999) and to suppress the MBN response to light during daytime (Saifullah and Tomioka 2002).

The current knowledge of the cricket’s circadian clock machinery is expected to further promote the use of crickets as model insects in chronobiology and other studies. One important aspect of such (already ongoing) work relates to the anthropogenic effects, specifically to light pollution.

## The cricket as a model for artificial light at night (ALAN) research

Light pollution, or artificial light at night (ALAN), is a constantly growing anthropogenic phenomenon (Hölker et al. 2010). ALAN disrupts various aspects of natural light, including its timing, duration, intensity, and spectrum (“color”) (Warrant and Johnsen 2013; Tamir et al. 2017), as well as the delicate balance of light and darkness (Aube 2015; Falchi et al. 2019; Garrett et al. 2020; Jechow et al. 2020), thus becoming a major environmental concern. Sources of ALAN include skyglow—with low light intensities of 0.07–1.1 lx and a very large area coverage (Kyba et al. 2011, 2012; Jechow et al. 2017, 2020; Hänel et al. 2018); streetlight illumination—with an intensity of ca. 2–10 lx and a large area coverage (Rich and Longcore 2006); and highly illuminated industrial areas and sports fields—with 1500 lx and above, constituting an intensity comparable to shaded daylight.

ALAN-induced obstruction of the natural light-dark cycle impacts the natural behavior of many animal and plant species, including humans, as well as whole ecosystems (Sanders and Gaston 2018; Garrett et al. 2020; Svechkina et al. 2020). In insects, ALAN results in temporal and spatial disorientation of ground-dwelling insects such as the dung beetle, and of flying insects such as moths and mayflies (Owens and Lewis 2018; Foster et al. 2021). Moreover, street lights that attract flying insects significantly increase insect mortality (Eisenbeis 2006; Perkin et al. 2014; Manfrin et al. 2017; Bolliger et al. 2020). ALAN can also affect the behavior of both predators and prey, leading to changes in food webs and ecosystem dynamics (Manfrin et al. 2017; Sanders and Gaston 2018; Baxter-Gilbert et al. 2021). Furthermore, ALAN induces a decrease in insect pollination (Knop et al. 2017; Borges 2018; Giavi et al. 2020), and changes in community structure and biodiversity (Sanders and Gaston 2018; Owens et al. 2020; Sanders et al. 2021). Further harmful consequences of ALAN include reduced immune reaction and impaired juvenile development in crickets (e.g., Durrant et al. 2020), and altered gene expression in glow-worms (Chen et al. 2021).

**Fig. 4:**
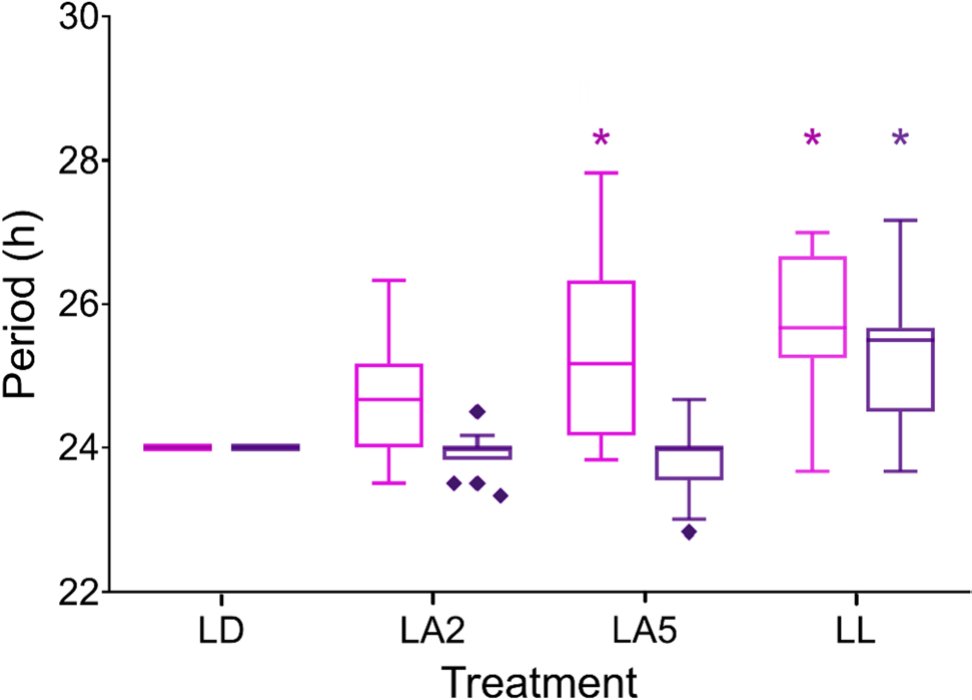
ALAN differentially affects the length of diurnal activity periods of stridulation (pink; n = 67) and locomotion (purple; n = 64) behaviors. Treatments: 12 h:12 h light: dark (LD), light:2 lx ALAN (LA2), light:5 lx ALAN (LA5), and 24 h constant light (LL). Tukey’s boxplot: Outliers (1.5 times the inter-quartile distance) are shown. Asterisks depict statistical significance from LD. Adapted from Levy et al. 2021, see details within

Crickets constitute a powerful model for studies of the effects of ALAN, and especially ecologically-relevant (dim) ALAN, as many crickets are nocturnal, presenting increased sensitivity to dim light, and their circadian behavior has been well-studied. Crickets in urban habitats are assumed to be exposed to lifelong ALAN. Consequently, Levy et al. (2021) experimentally exposed field crickets (*G. bimaculatus*) to different lifelong illumination conditions. Simultaneous monitoring of stridulation and locomotion in individual males was utilized to assess the possible effect of lifelong dim-ALAN on each of these behaviors independently, as well as in comparison to one another. In undisturbed (control) crickets, stridulation was predominantly nocturnal and locomotion behavior was diurnal. However, the temporal differences between nocturnal and diurnal behavior diminished with increasing ALAN intensity. Moreover, the percentage of individuals showing free-run behavior (Fig. 2b) increased with increasing lifelong ALAN intensity, leading to behavioral desynchronization of the population. Lastly, changes in the medians of the daily activity periods were found to differ for both stridulation and locomotion (Fig. 4). This may have been the result of a masking response in the diurnal locomotion, or it may indicate differential susceptibility of the different behaviors to the same light stimulus (Levy et al. 2021).

In a follow-up study by Levy et al. (2022) the molecular correlates of ALAN were recorded. Crickets were subjected to a short, dim ALAN pulse during their early subjective night. The relative expression of five circadian clock-associated genes was compared in four tissues, including the brain and optic lobe. An analysis of their relative transcriptional responses revealed two clearly separate responses in these two tissues. In the brain, the expression of *cry2, per*, and *opLW* increased with increasing light intensity; while in the optic lobe an overall decrease in expression was observed (with the exception of an increase in *opLW*). These tissue, gene, and light-intensity related effects reconfirm the relation between immediate transcriptional response and circadian behavior.

Revisiting the differential effects of light on the cricket’s stridulation and locomotion behaviors, Levy et al. (2023a) exposed male crickets to a nocturnal light pulse, similar in intensity to their previously utilized experimental daylight (40 lx). Their findings revealed a simultaneous negative masking of stridulation (transiently reducing the behavior), and a positive masking of locomotion (an increase towards daytime levels; Fig. 2c). Thus, both a transient and a lifelong exposure to ALAN may affect the timing and pattern of both these behaviors. Notably, stridulation serves for attracting potential mates, while locomotion is used for foraging. Consequently, the ALAN-induced behavioral changes may negatively impact the crickets’ reproductive success and fitness and thereby increase the vulnerability of the population.

The crickets’ immune responses are also affected by ALAN. A reduced cellular immune response (haemocyte concentration) in the black field cricket, *T. commodus*, was described following exposure to ALAN (Durrant et al. 2015, 2020). Moreover, the effect of lifelong exposure of these crickets to dim-ALAN as low as 1 lx was similar to the effects induced by 10 and 100 lx. Hence, even dim ALAN presents malign conditions, impacting the cricket’s immune response (Durrant et al. 2020) and potentially perceived as constant light by the nocturnal, light-sensitive crickets.

In most of the animals studied to date (e.g., Haim et al. 2015; Raap et al. 2015; Vivien-Roels et al. 1984), the level of melatonin was reported to follow the day-night rhythmicity and to strongly decrease following exposure to light (also reported to be reflected in the compound eyes and brain of crickets; Itoh et al. 1995). Melatonin is an antioxidant thought to be related to the immune system (Carrillo-Vico et al. 2013; Melendez-Fernandez et al. 2023). Accordingly, the crickets’ light-induced degraded immune response has been suggested to be melatonin-related (Durrant et al. 2015; Jones et al. 2015). The possible role of melatonin in light-induced effects in the cricket awaits, however, further research.

Crickets have also constituted a valuable model for outdoor experiments and field studies such as studies of population dynamics in natural settings (Tregenza 2003; Bretman et al. 2008; Fisher et al. 2019), as well as studies of the impact of anthropogenic noise pollution on intraspecific communication (Costello and Symes 2014; Duarte et al. 2019; Tanner and Simmons 2022). Recently, the effect of ALAN on stridulation behavior of *G. bimaculatus* crickets was studied under semi-natural conditions (Levy et al. 2023b). Adult male crickets were individually housed in shaded enclosures in their natural habitat and their behavior was acoustically monitored for two consecutive weeks, while exposing them to different ALAN regimes. The findings revealed an ALAN-intensity-dependent increase in the percentage of individuals that exhibited free-run behavior, along with a corresponding increase in the activity periods. These findings support the conclusion that ALAN may ultimately lead to a desynchronizing effect on the population. Notably, the threshold at which 80% of individuals exhibited free-run behavior was considerably higher in the semi-natural settings, compared to laboratory conditions. This discrepancy may however primarily relate to the overall differences between the indoor and outdoor conditions, specifically to the more intense natural diurnal light as well as the natural temperature rhythms (Levy et al. 2023b, and see below).

## Further comments and challenges

In contrast to the extensively studied circadian clock of crickets, our comprehension of the ecologically-relevant light effects on these insects remains incomplete. Specifically, it is important to fill in the existing knowledge gaps between sensory perception, circadian gene expression, and behavioral processes. Currently, there is a dearth of knowledge regarding the distinct light thresholds required to elicit a response in the compound eye; the functioning of the circadian clock mechanism; and the influence of light on specific, and perhaps all, behaviors examined to date. Furthermore, these thresholds and responses may vary both among individuals of the same species and between different species. As noted previously, a comprehensive understanding of the effects of specific physical properties of the light stimulus is still lacking and warranted. Beyond the above-noted behavioral patterns, it is conceivable that light may also play a role in modulating other critical behaviors, such as hatching, molting, navigation, and more.

The differing responses to environmental illumination cues underline the complexity of the circadian system, specifically when comparing entrainment and masking. Under light-dark conditions the exposure of crickets to a nocturnal 3-h light pulse evoked an immediate masking response (Germ and Tomioka 1998; Levy et al. 2023a), inducing a simultaneous decrease and even cessation of stridulation (negative masking), while increasing locomotion (positive masking) (Levy et al. 2023a). However, under constant darkness a 3-h light pulse was reported to phase shift the circadian clock (Okada et al. 1991; Kutaragi et al. 2016). Moreover, repetitive 15-min light pulses under constant darkness were reported to induce rhythm synchronization, depending on the interval between the pulses (Germ and Tomioka 1998). Hence, the type and extent of the response to light stimuli depend on the surrounding illumination context, intensity, timing, duration, repetitivity, and intervals of the stimuli. Further research on this remarkable complexity of the circadian clock may be of importance in assessing the possible short- or long-term effects of various outdoor illumination, such as car headlights or streetlights, on nocturnal insects. Reducing the duration of outdoor illumination may be helpful in protecting nocturnal insects.

Current research also aims at understanding the impact of environmental temperature. Many organisms in their natural habitat are exposed to a wide range of thermoperiods and temperature fluctuations. However, their period of daily activity rhythm has been reported to remain stable, demonstrating the remarkable property of temperature compensation (Aschoff 1981; Saunders et al. 2002). This was recently confirmed in crickets, which also demonstrated stable activity periods despite temperature fluctuations and seasonal changes (Levy et al. 2023b).

Temperature cycles, acting as entraining agents (*zeitgebers*) in insects, including crickets, have been reported under constant darkness (Loher and Wiedenmann 1981; Saunders et al. 2002; Beer and Helfrich-Förster 2020). For instance, crickets rendered arrhythmic following optic lobe removal regained rhythmic stridulatory activity under constant light when exposed to a daily cycle of 12 h hot and 12 h cold temperature, stridulating during the cold phase (Rence and Loher 1975). In *T. commodus*, a coupled light- and thermoperiod system showed distinctive patterns, with thermoperiod entrainment displaying higher percentages of entrainment with larger temperature oscillations (Rence 1984). Temperature cycles also influenced locomotor behavior and clock gene transcriptional rhythms in *G. bimaculatus* (Kannan et al. 2019).

Despite these findings, light appears to be the primary *zeitgeber* (Rence and Loher 1975; Kannan et al. 2019; Beer and Helfrich-Förster 2020). A 14-day outdoor experiment by Levy et al. (2023b) revealed a strong ALAN-induced free-run pattern, despite near-natural thermoperiods. The absence of temperature entrainment could be attributed, as suggested by Rence (1984), to initially introducing crickets to LD conditions and later to almost natural thermoperiods. Rence emphasized the importance of temperature signals and the order of *zeitgebers* (light and temperature). Additionally, temperature entrainment may require more cycles than light entrainment (Rence 1984; Kannan et al. 2019). The interplay of light and temperature as *zeitgebers* in crickets, highlighting the complex nature of the circadian system, warrants further investigation.

In summary, exploring the full impact of light on the circadian behavior of the cricket (including the significant negative ecological impact of ALAN) requires an interdisciplinary, multi-modal approach, incorporating the insect’s visual system, its relevant illumination thresholds, and its sensitivity to physical light properties, the circadian clock mechanism, and behavior. Such an approach will deepen our understanding of the major role of light on natural habitats and its effect on various aspects of animals’ fitness, reproduction, and dispersal.

### Supplementary Information

Below is the link to the electronic supplementary material.

Supplementary material 1 (MP4 38880.0 kb)
